# Influence of brain-derived neurotrophic factor on pathfinding of dentate granule cell axons, the hippocampal mossy fibers

**DOI:** 10.1186/1756-6606-2-2

**Published:** 2009-01-31

**Authors:** Makoto Tamura, Naohiro Tamura, Takamitsu Ikeda, Ryuta Koyama, Yuji Ikegaya, Norio Matsuki, Maki K Yamada

**Affiliations:** 1 Laboratory of Chemical Pharmacology, Graduate School of Pharmaceutical Sciences, The University of Tokyo, 7-3-1 Hongo, Bunkyo-ku, 113-0033, Tokyo , Japan; 2 PRESTO, JST, Honcho, Kawaguchi-shi, Saitama 332-0012, Japan

## Abstract

Mossy fibers, the dentate granule cell axons, are generated throughout an animal's lifetime. Mossy fiber paths and synapses are primarily restricted to the stratum lucidum within the CA3 region. Brain-derived neurotrophic factor (BDNF), a neurotrophin family protein that activates Trk neurotrophin receptors, is highly expressed in the stratum lucidum in an activity-dependent manner. The addition of a Trk neurotrophin receptor inhibitor, K252a, to cultured hippocampal slices induced aberrant extension of mossy fibers into ectopic regions. BDNF overexpression in granule cells ameliorated the mossy fiber pathway abnormalities caused by a submaximal dose of K252a. A similar rescue was observed when BDNF was expressed in CA3 pyramidal cells, most notably in mossy fibers distal to the expression site. These findings are the first to clarify the role of BDNF in mossy fiber pathfinding, not as an attractant cue but as a regulator, possibly acting in a paracrine manner. This effect of BDNF may be as a signal for new fibers to fasciculate and extend further to form synapses with neurons that are far from active BDNF-expressing synapses. This mechanism would ensure the emergence of new independent dentate gyrus-CA3 circuits by the axons of new-born granule cells.

## Background

Mossy fibers, which are the dentate granule cell axons, are the only excitatory efferent projections of the hippocampal dentate gyrus, which has a crucial role in certain types of learning and memory. Mossy fibers have several unique features. Each mossy fiber forms extraordinarily large synapses with the proximal apical dendrites of only 11 to 18 pyramidal cells in the CA3 region [1]; further, the action potentials of a single granule cell are strong enough to discharge its target neurons. Importantly, granule cells are continuously produced, even in adulthood [2], and newly formed granule cells in the adult brain are more likely to be recruited into circuits related to spatial memory than existing granule cells [3]. Thus, mossy fibers from newly formed granule cells are likely influenced by neuronal activity. Elucidation of the neuronal activity-related nature of these fibers, therefore, may provide clues to the *raison d'etre *of these peculiar axons and dentate granule cells.

Mossy fibers bundle tightly together and extend into a narrow region of the CA3 region called the stratum lucidum (SL), where they form synapses with CA3 pyramidal cells. Inhibition of Trk neurotrophin receptors and the Trk downstream molecule mitogen-activated protein (MAP) kinase kinase (MEK) disrupts mossy fiber pathfinding [4]. The Trk receptors, TrkA, TrkB, and TrkC, are primarily activated by nerve growth factor (NGF), brain-derived neurotrophic factor (BDNF), and neurotrophin-3, respectively. Because the highest BDNF protein levels in the brain are found along the pathway from the dentate hilus to the SL [5,6], BDNF is the candidate molecule most likely to be involved in regulating mossy fiber pathfinding. Moreover, the activity-dependence of BDNF biosynthesis and secretion [7,8]. raises the interesting possibility that BDNF mediates the interaction between active and newly-formed BDNF-expressing neuronal circuits.

Several studies have focused on BDNF-induced abnormalities in mossy fiber sprouting, which may be one etiology of temporal lobe epilepsy. BDNF, which is highly upregulated in some hyperactivity conditions, may [9,10] or may not [11,12] induce mossy fiber sprouting [13]. The role of BDNF in mossy fiber pathfinding under normal conditions remains to be elucidated.

To investigate the role of BDNF in healthy mossy fiber formation, we applied the broad-spectrum Trk neurotrophin receptor inhibitor K252a in combination with local BDNF gene expression induced with a lentivirus, which produced moderate and physiologic levels of BDNF. This combined molecular and pharmacologic approach allowed us to selectively examine effects of BDNF that may be overlooked with simple loss-of-function experiments, given the potential for redundancy between BDNF and NGF within the SL.

## Results

### Gene expression and development of an assay system for tracking mossy fiber pathfinding

**Figure 1:**
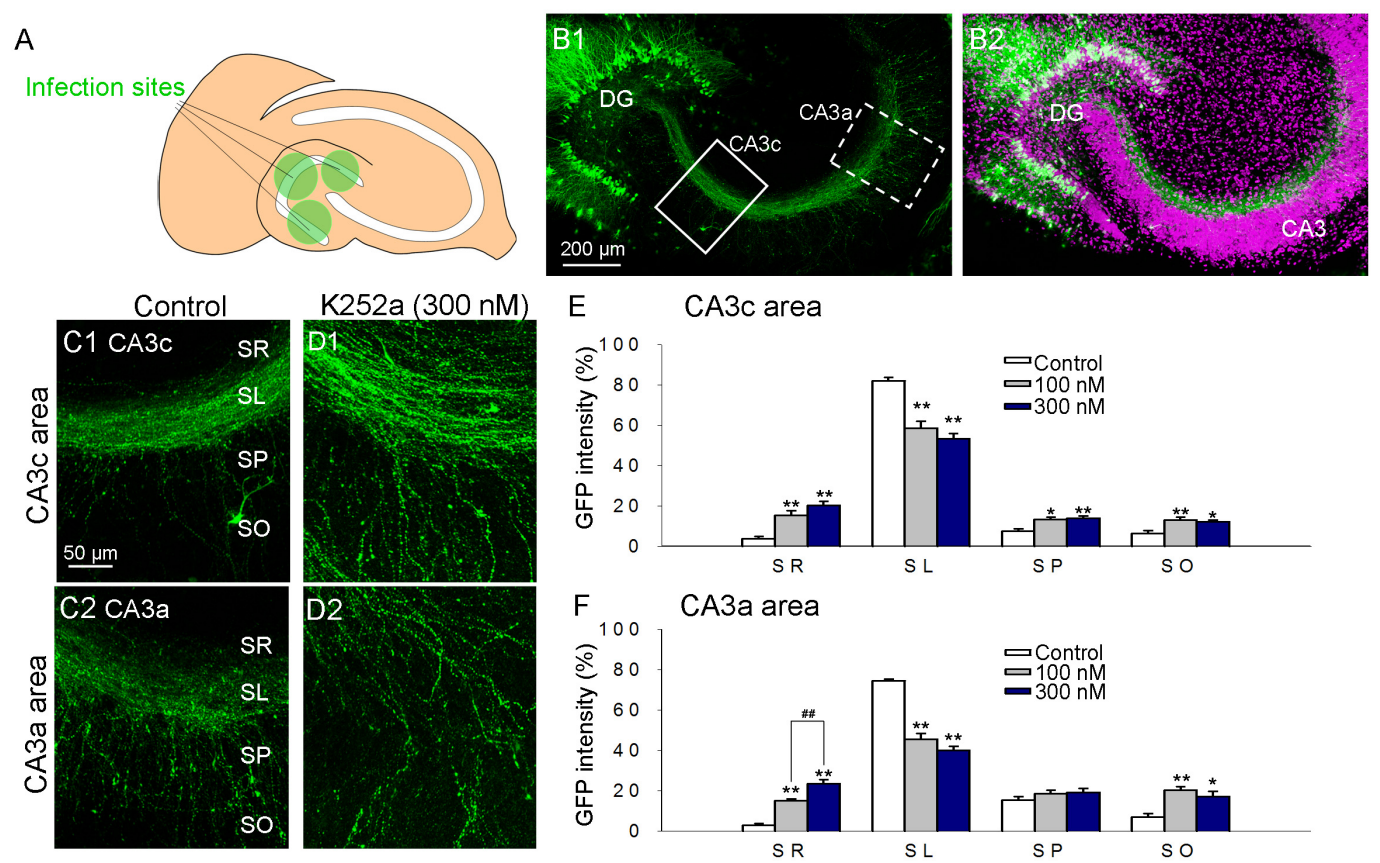
**A novel approach for visualizing mossy fibers in cultured hippocampal slices**. (A) A schematic diagram of locally introduced lentiviral vectors. Lentivirus, which induces the expression of enhanced GFP (EGFP) under the influence of a CaMKII promoter, was injected into 2 or 3 sites of the granule cell layer in hippocampal slices. (B) Representative images of the results. Granule cells specifically expressed EGFP. Green: EGFP immunoreactivity, Magenta: Nissl staining of the neurons. (C and D) Images of control slices (C) and slices treated with K252a (300 nM) (D) in the CA3c (C1, solid box in B1) and CA3a (C2, dotted box in B1) areas. Mossy fibers in control slices (C) were observed primarily in the SL, similar to what was observed *in vivo*, while those in the K252a-treated slices were abnormally distributed outside of the SL as shown in D. (E and F) Distribution of the mossy fibers treated by 100 and 300 nM of K252a as assessed by the measurement of EGFP intensity in the CA3c (E) and CA3a (F) areas. *P < 0.05 and ^##^, **P < 0.01; Tukey's test after analysis of variance (n = 7–9 slices obtained from 4 independent experiments). DG: dentate gyrus, SR: stratum radiatum, SP: stratum pyramidale, and SO: stratum oriens.

**Figure 2:**
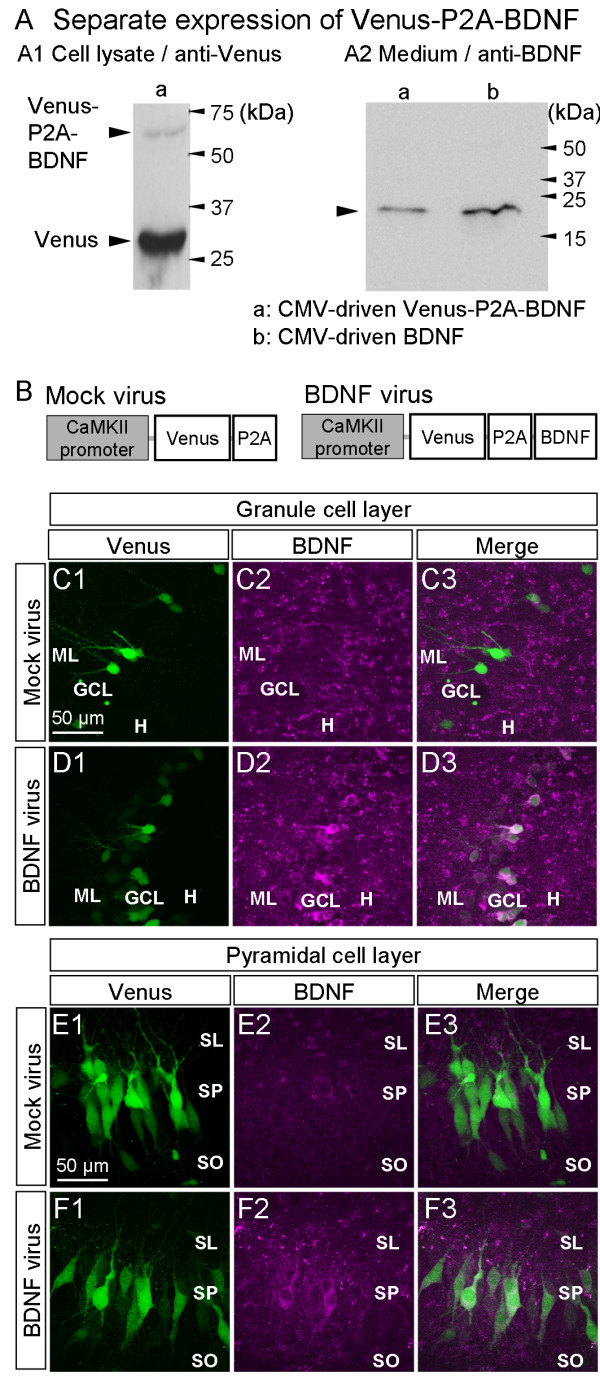
**Co-expression of BDNF and Venus using P2A peptide**. (A) Protein expression with the Venus-P2A-BDNF construct, which was designed to produce Venus-P2A fusion protein and BDNF separately, was confirmed by performing an immunoblotting analysis for Venus, a GFP mutant, (A1) or BDNF (A2). In this experiment, the Venus-P2A-BDNF construct was transfected to HEK293T. Even though a weak band was noted at the position of the non-cleaved fusion protein for Venus-P2A-BDNF, almost all signals were detected for the cleaved Venus-P2A. (A2) In the culture medium of HEK293 cells transfected with the Venus-P2A-BDNF construct (lane a), a specific band was observed at the same molecular weight as that for the control from cells showing BDNF expression (lane b); this indicated that BDNF, when cleaved from the Venus-P2A-BDNF construct, followed the normal course of processing and secretion. (B) Schematic diagrams of the expression vectors. (C-F) Immunohistochemical staining for Venus (green) and BDNF (magenta) in the dentate gyrus (C, D) and CA3 region (E, F) of cultured slices transfected with the mock Venus-P2A- (C, E) or Venus-P2A-BDNF-carrying (D, F) virus. In D and F, Venus-positive cells showed intense BDNF staining in slices transfected with the Venus-P2A-BDNF virus, whereas endogenous BDNF and Venus were rarely colocalized in slices transfected with the mock virus (C and E). ML: molecular layer, GCL: granule cell layer, DH: dentate hilus, SL: stratum lucidum, SP: stratum pyramidale, and SO: stratum oriens.

A neuron-specific α-calmodulin-dependent protein kinase II (CaMKII) promoter was used for all expression experiments [14]. The lentivirus suspension, which expresses enhanced green fluorescent protein (EGFP), was locally introduced at 2 or 3 sites in the granule cell layer in hippocampal slices; almost all cells expressing EGFP were granule cells, based on their cellular morphology and localization in the granule cell layer (Fig. 1A and 1B, Fig. 2C and 2D). The mossy fiber pathways could be specifically and clearly visualized by this method (Fig. 1B–D). At 8 days *in vitro *(DIV), EGFP-expressing mossy fibers in the control slices were generally restricted to the normal path, the SL (Fig. 1C). In K252a-treated slices, however, mossy fibers were abnormally distributed outside the SL in both the CA3c and CA3a areas (Fig. 1D). In these slices, the EGFP signal intensity was decreased in the SL and increased in subregions other than the SL (Fig. 1E and 1F). These data indicated that our new assay system was able to produce and detect the aberrant mossy fiber growth induced by K252a that was reported previously [4].

### Co-expression of BDNF and Venus using the P2A peptide

Mossy fiber pathways in the cultured hippocampal slices were rich in BDNF (Additional file 1), consistent with previous findings in brain sections [15]. Thus, BDNF was selected from among candidate molecules whose signals are attenuated by K252a. BDNF overexpression was induced by lentivirus-mediated gene transfer to determine whether the effects of a submaximal dose of K252a could be attenuated. To identify cells overexpressing BDNF, we co-expressed Venus, a GFP variant, using a self-cleaving 2A sequence derived from picornavirus (P2A). Through a ribosomal "skip" mechanism, the P2A peptide interconnecting two different proteins spontaneously cleaves, thereby separating the proteins and theoretically producing equivalent amounts of both proteins [16].

A Venus-P2A-BDNF sequence was constructed by inserting the P2A sequence between the Venus and BDNF sequences. Distinct expression of BDNF and Venus was confirmed by immunoblotting analysis of HEK293T cell lysates transfected with the Venus-P2A-BDNF construct. We confirmed that the expressed BDNF in the HEK293T cells was secreted into the culture medium (Fig. 2A).

The BDNF expression levels produced in the cultured slices using this virus were similar to the BDNF levels that are endogenously expressed and accumulated, and were visualized as punctate staining (Fig. 2B–F). We did not observe the growth of any aberrant basal dendrites or axonal branches as has previously been observed in BDNF-overexpressing cells produced using a Gene gun [17]; this discrepancy may be due to the fact that our experimental system produced close to physiologic levels of BDNF expression.

### BDNF-overexpressing mossy fibers find normal pathways in the presence of a submaximal dose of K252a

**Figure 3:**
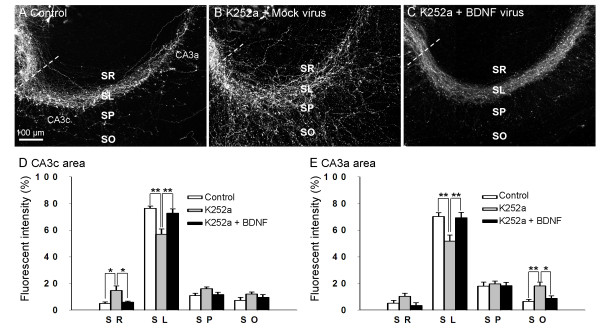
**BDNF expressed in granule cells rescued the aberrant mossy fiber extension induced by a submaximal dose of K252a (100 nM)**. (A-C) Compared with control slices (A), K252a-treated slices infected with mock viruses showed an abnormal distribution of mossy fibers (B). Meanwhile, the BDNF-expressing mossy fibers extended into the SL (C) in a manner similar to that observed in the control slices. The dotted line indicates the incision line made 4 days after transfection to adjust the axonal extension to the expression of proteins. (D and E) Mossy fiber distribution analyzed by a ratio distribution of the co-expressed Venus in the CA3c (D) and CA3a (E) areas. *P < 0.05 and **P < 0.01; Tukey's test after analysis of variance (n = 7–9 slices obtained from 4 independent experiments). SR: stratum radiatum, SP: stratum pyramidale, and SO: stratum oriens.

In the expression experiments, 4 days after transfection, preexisting mossy fibers were cut at a site proximal to the CA3 region (dotted lines in Fig. 3), and the paths of newly extended fibers expressing Venus were observed, because substantial BDNF expression was obtained by 4 DIV (data not shown). A 7-day K252a treatment was initiated soon after the incision. In the control (nonK252a-treated) slices, the Venus-labeled mossy fibers normally reextended to and within the SL 7 days after the fibers were cut (Fig. 3A). While treatment with low dose (100 nM) of K252a was enough to defasciculate mossy fibers in the ectopic subregions (Fig. 3B), similar to that shown in Figure 1, BDNF-overexpressing mossy fibers extended to the SL (Fig. 3C), similar to that observed in control (nonK252a-treated) slices (Fig. 3D and 3E).

In order to determine the dose-response relationship between the concentration of K252a and the action of BDNF, we performed the same experiments with increasing doses of K252a. We observed that the slices treated with higher concentrations of K252a (200 nM and 300 nM) were rarely affected by BDNF expression (Additional file 2).

These findings indicate that BDNF secreted by granule cells (mossy fibers) supports normal mossy fiber pathfinding.

### BDNF overexpression in CA3 pyramidal cells also rescues abnormal mossy fiber projection

**Figure 4:**
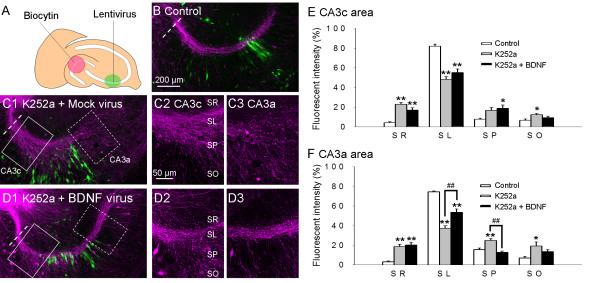
**Overexpression of BDNF in CA3 pyramidal cells promotes mossy fiber pathfinding in the distal parts of the axons**. (A) The expression viruses, including the CaMKII promoter were injected locally in the CA3 cell layer (green circle). Biocytin crystals were placed on the granule cell layer (pink circle) to visualize mossy fibers. (B) In nontreated control slices, mossy fibers visualized by the biocytin labeling (magenta) were fasciculated, and CA3 cells infected with mock viruses were identified by the co-expression of Venus (green). (C and D) K252a-treated slices with mock (C) or BDNF (D) expression are shown in high magnification images of the CA3c (2: solid box in 1) or CA3a (3: dotted box in 1) area. D shows abnormal distribution of mossy fibers in the CA3c area; these fibers began to accumulate in the CA3b area, which contained pyramidal cells overexpressing BDNF (green), and fasciculated tightly in the SL of the CA3a area. (E and F) Mossy fiber distribution estimated by biocytin labeling intensity in the CA3c (E) and CA3a (F) regions. *P < 0.05 and **, ^##^*P *< 0.01; Tukey's test after analysis of variance (n = 9 slices obtained from 4 independent experiments). SR: stratum radiatum, SP: stratum pyramidale, and SO: stratum oriens.

To determine the possible site and mode of BDNF action, we investigated whether BDNF overexpressed by CA3 pyramidal cells also regulates mossy fiber pathfinding. Visualization of mossy fibers using biocytin (Fig. 4A and, 4B) confirmed the presence of misguided and defasciculated fibers in the K252a-treated slices (Fig. 4C). In the presence of K252a, when BDNF-carrying viruses were injected in the CA3b area at the middle of the CA3 region, mossy fibers extended beyond the CA3b area to the more distal CA3a area and the paths gradually bundled within the SL (Fig. 4D–F).

Structures like presynaptic varicosities were observed in the labeled mossy fibers. It is noteworthy, however, that the passing fibers did not form obvious varicosities on any BDNF-expressing CA3 neurons (Fig. 4D).

## Discussion

In this study, we used a combined molecular and pharmacologic approach to examine the effect of BDNF on hippocampal mossy fiber pathfinding. Rat brain hippocampal slices that included the CA3 region were treated with a noncompetitive inhibitor of tyrosine kinase activity of Trk receptors, K252a [18], combined with lentiviral vector-induced BDNF overexpression in either granule cells or pyramidal cells. K252a, which was used to inhibit the possible action of neurotrophin receptors, was used at a submaximal dose (Fig. 1D and Additional file 2) and is thus considered to act partially, allowing for the remaining receptors to be activated by the expressed BDNF. The results indicated that the overexpressed BDNF in both types of cells rescued the K252a-induced abnormal mossy fiber pathfinding.

BDNF is a neurotrophin that activates the Trk receptor family, and other neurotrophins are suggested to have compensatory roles [19]. Therefore, although BDNF-deficient mice have normal mossy fiber paths (Tamura et al., unpublished observation) and TrkB-deficient mice do not show aberrant defasciculation of mossy fibers [20], BDNF may still be critical for mossy fiber pathfinding because other neurotrophins, such as NGF, that are also expressed in the mossy fiber pathways [20], may apparently compensate for the lack of BDNF signaling.

Experiments with pharmacologic inhibitors sometimes overcome functional redundancy in molecules. In addition, gene expression (of BDNF in this case) is a powerful method to confirm the specificity of molecular participants compared to pharmacologic treatment. In the present study, a novel combination of pharmacologic and molecular expression approaches was developed to elucidate the function of a molecule that may have otherwise remained hidden due to redundancy. Although rescue experiments using multiple gene-deficient mice, as reported previously [21], may be more powerful, the use of the present combined molecular and pharmacologic approach demonstrated for the first time that BDNF influences mossy fiber pathfinding. A similar combinatorial approach may be a useful first step toward examining the biologic functions of molecules within a family.

Although BDNF functions as a chemoattractant for various neurons, including *Xenopus *spinal neurons, rat cerebellar neurons, and chick retinal ganglion neurons [22-24], the present findings in the hippocampus were not consistent with a chemoattractant function of BDNF for the following reasons. First, BDNF produced within the guided axons was sufficient to regulate their paths (Fig. 3); and second, when BDNF was expressed in CA3 pyramidal cells, the fibers did not target the cells in which BDNF was overexpressed, but rather gradually became fasciculated after they passed through the region of cells overexpressing BDNF (Fig.4). BDNF may instead affect the expression and/or activation of cell adhesion molecules that form mossy fiber bundles with one another and/or bind to the proximal dendrites of CA3 pyramidal cells. Reports showing that BDNF mediates cadherin-catenin interactions in dissociated hippocampal neurons and that some types of cadherin-deficient mice have abnormal mossy fiber projection might be consistent with our hypothesis of the underlying mechanism [25,26].

Alternatively, BDNF might induce the activation of receptors for repulsive guidance factors secreted from subregions other than the SL, such as the stratum radiatum and stratum pyramidale. Semaphorin 3s [27], Semaphorin 6A [28], and Slit-2 [29] function as repellents for mossy fibers. BDNF may act as a key regulator of other molecules by inducing and modulating their function.

The activity-dependent nature of BDNF [30] may be fundamentally associated with its neurobiologic functions. Interestingly, Danzer et al. (2004) showed that BDNF immunoreactivity is detected in mature presynaptic boutons of mossy fibers, and that the number of BDNF-rich presynaptic boutons is increased by pilocarpine-induced neuronal activity [7]. In our overexpression experiments, the artificially expressed BDNF would mimic that in mature mossy fiber terminals, thus it is likely that endogenous BDNF has a paracrine effect on newly generated fibers. It might be possible that the pathfinding effect of BDNF is involved in the activity-dependent plasticity underlying memory mechanisms. It would not, however, directly and immediately contribute to these mechanisms because the time course of mossy fiber pathfinding examined in this study (over several days) appears to be too long and newly-originated mossy fibers require 2.5 weeks to mature in adult mice [31]. Rather, the effect of BDNF may be as a signal for new fibers to fasciculate and extend further into the SL to form synapses with neurons that are far from active BDNF-expressing synapses. This view is supported by our finding that the passing fibers did not form obvious synaptic varicosities on any of the BDNF-expressing CA3 neurons (Fig. 4D). This mechanism would ensure the emergence of new independent dentate gyrus-CA3 circuits, which are hypothetically required to effectively encode information similar to, but distinct from, the preexisting circuits [32]. Dysregulation would cause information overwriting, which might be a clue to why schizophrenia has been related to BDNF reduction [33,34]. Recent suggestions that the etiology of schizophrenia may be due to adult neurogenesis [35] and subsequent mossy fiber guidance [36] seem to be consistent with this hypothesis.

## Conclusion

Our findings are the first to clarify the role of BDNF in mossy fiber pathfinding; BDNF functions not as an attractant cue but as a regulator, possibly acting in a paracrine manner. This effect of BDNF may be as a signal for new fibers to fasciculate and extend further to form synapses with neurons that are far from active BDNF-expressing synapses. This mechanism would ensure the emergence of new independent dentate gyrus-CA3 circuits by the axons of new-born granule cells.

## Methods

### Organotypic cultures of hippocampal slices

Entorhino-hippocampal slices (300 μm thick) were prepared as previously described [9]. In some slices, the mossy fibers were cut with a scalpel after 4 DIV [37]. Slices were cultured using a membrane interface technique (Millicell-CM, Millipore, Bedford, MA) [38]. Cultures were fed 1 ml of culture medium consisting of 50% minimal essential medium (Sigma, St. Louis, MO), 25% horse serum (Cell Culture Lab, Cleveland, OH), and 25% Hanks' balanced salt solution containing 25 mM glucose, 50 units ml^-1 ^penicillin G, and 100 μg ml^-1 ^streptomycin, and maintained in a humidified incubator at 37°C in 5% CO_2_. The medium, with or without K252a (Wako, Osaka, Japan), was changed every 3.5 days.

### Immunohistochemistry and histology

Cultured hippocampal slices were immersed in 4% paraformaldehyde at 4°C for 4 h and treated with 0.3% Triton X-100 for 12 h. After a 1-h incubation in 5% goat serum at room temperature, rabbit anti-GFP antibody (1:1000; Molecular Probes, Eugene, OR) or anti-BDNF antibody (0.5 μg/ml, a generous gift from Amgen) in phosphate buffered saline (PBS) containing 2% goat serum was applied at 4°C for 24 h followed by Alexa Fluor 488 goat anti-rabbit secondary antibody (1:500, Molecular Probes) for 4 to 6 h, or biotinylated anti-rabbit secondary antibody (Vector Laboratories, Burlingame, CA) for 1 h at room temperature. To visualize Venus signals in mossy fibers, the TSA fluorescein system (PerkinElmer Life, Boston, MA) was used with 1-h incubation in avidin-biotin complex solution (Vector Laboratories) following treatment with anti-GFP antibody and quenching of endogenous peroxidase activity with H_2_O_2 _(0.3%) for 20 min.

For biocytin labeling, biocytin crystals were placed on the granule cell layer of cultured slices 4 h before fixation. These slices were fixed and permeabilized as for immunohistochemistry, and finally incubated with 10 μg/ml Texas Red-X-conjugated streptavidin (Molecular Probes) in a dark room at room temperature for 4 h. For Nissl staining, slices were immersed in PBS containing 4% paraformaldehyde for 24 h, washed three times with PBS, treated with 0.1% Triton-X-100 for 60 min, washed, and then incubated with NeuroTrace fluorescent Nissl (1:100 dilution; Molecular Probes) for 6 h at room temperature, followed by three rinses with PBS. All fluorescence signals were observed using a MRC-1000 confocal imaging system (Bio-Rad) with a 20× objective lens. Mossy fiber distribution was assessed by analyzing the EGFP or Venus signal ratio in each CA3 subregion. The average intensities of the fluorescent signals were quantified in five randomly selected areas (10 μm × 10 μm) of each subregion. The subregions were defined on the basis of the distance from the stratum pyramidale (layer of soma), i.e., the SL is the region within 70 μm from the stratum pyramidale, the stratum radiatum is the region above the SL, and the stratum oriens is beyond the stratum pyramidale. The signal intensity was normalized using the sum of the raw intensities of all four CA3 subregions.

### Construction of the BDNF-expressing lentivirus vector

To produce the cDNA coding for Venus, a brighter GFP derivative was provided as a kind gift from Dr. Miyawaki [39] and modified by PCR. The P2A sequence, a porcine teschovirus-1-derived 2A peptide sequence, includes a 2A consensus signal, D (V/I) EXNPG-P. The 2A signal is associated with a ribosomal skip in the peptide-bond formation between the glycine residue and the following proline residue [16].

BDNF cDNA capped with the Kozak sequence was connected in-frame with the Venus-P2A sequence through the BamHI site. The resultant BDNF was logically capped by PGST peptide at the N-terminus and the functionality of the secretion signal was confirmed experimentally (Fig. 2A2). The final BDNF protein was tagged with human c-Myc peptide, QKLISEEDLNGAA at the C-terminus, and its activity has been confirmed [40]. The control mock virus plasmid has a stop codon immediately downstream of the BamHI site.

### Lentivirus preparation

A lentiviral vector, FCGW, was a generous gift from Dr. P.V. Osten [14]. Lentiviruses were produced by HEK293T cells co-transfected with the expression vector (3 μg) and three helper plasmids in a mixed package (9 μg; Invitrogen) using Lipofectamine 2000 (36 μl per 10-cm culture dish). The medium was changed once after 18 to 24 h. Seventy-two hours after transfection, the medium was spun at 780 × *g *for 15 min, passed through a 0.45-μm filter and centrifuged at 83,000 × *g *for 90 min, then the pellet was resuspended in 100 μl medium. The final titer was approximately 10^8 ^PFU/ml. Dissociated hippocampal cultures were exposed to the final suspension and then analyzed by immunocytochemistry for EGFP.

### Microinjection of vector suspension into the hippocampal slice cultures

Micropipettes made from glass capillaries were autoclaved and the tips were broken to obtain a diameter of approximately 0.5 μm. For each experiment, a pipette was filled with a few microliters of lentiviral-vector suspension and placed directly over the cell layer using a micromanipulator (Narishige, Tokyo, Japan) and microejected using a brief (10–20 ms) pulse of compressed N_2 _gas via a Picoinjector (PLI-100; Narishige; 3.5–4.0 p.s.i). The suspension that was successfully delivered onto the slice was visually confirmed under the microscope. The BDNF virus or mock virus infection procedures were performed soon after the slices were prepared. Because lentivirus-mediated gene delivery expression plateaus 4 d after virus infection (data not shown), mossy fibers were cut once at 4 DIV. After the incision, a submaximal dose of K252a (100 nM) was applied and left in place for 1 week, during which mossy fiber regeneration was allowed to proceed [41].

### Statistics

Data are expressed as means ± SEM values. Tests of variance homogeneity, normality, and distribution were performed to ensure that the assumptions required for a standard parametric ANOVA were satisfied. Statistical analysis was performed by Student's *t*-test for two pair-wise comparisons and one-way repeated-measures ANOVA and *post hoc *Tukey's test for multiple pair-wise comparisons. Significance was set at the *P *< 0.05 level.

## Competing interests

The authors declare that they have no competing interests.

## Authors' contributions

MT performed all experiments except as otherwise noted with his original ideas of experimental details and wrote the manuscript draft. NT contributed on making a part of viral vector construct. TI carried out the expression in HEK and immunobloting. YI set up and RK developed slice culture experimental system. NM coordinated experimental environment as a professor. YI, RK and NM partly participated in the supervision of the work. MKY is conceived of research design, constructed the plasmid inserts for virus, wrote the manuscript and supervised. All authors helped to draft the manuscript.

## Supplementary Material

Additional file 1 **Endogenous BDNF was expressed primarily along the mossy fiber pathways in cultured hippocampal slices, as observed in brain section**s. Merged images of hippocampal slices stained (C, merged) with both Nissl staining of neurons (B) and BDNF immunohistochemistry (A). BDNF was highly expressed in the SL (arrow) and the dentate hilus, which are the normal mossy fiber pathways. GCL: granule cell layer, PCL: pyramidal cell layer, and DH: dentate hilus. Click here for file

Additional file 2 **Higher doses of K252a defasciculated the BDNF-overexpressing mossy fibers**. We performed the same experiments as in the case of figure 3; however, we used higher doses [200 nM (A) and 300 nM (B)] of K252a. Mossy-fiber distribution was analyzed by determining the intensity of coexpressed Venus in the CA3c area (left) and the CA3a area (right). We observed a minor effect of the expressed BDNF at 200 nM (A); however, the effect was much lower than that observed in case of 100 nM K252a, suggesting that BDNF could overcome the effect of K252a at submaximal doses and that BDNF action on mossy fibers is dependent on Trk signaling. *P < 0.05 and **P < 0.01; Tukey's test after analysis of variance (n = 7–9 obtained from three independent experiments). SR: stratum radiatum, SP: stratum pyramidale and SO: stratum oriens. Click here for file
