# Supplementary figures and images for: Influence of brain-derived neurotrophic factor on pathfinding of dentate granule cell axons, the hippocampal mossy fibers
Source: Mol Brain. 2009 Jan 31;2:2. doi: 10.1186/1756-6606-2-2 (PMC2642816; doi:10.1186/1756-6606-2-2)

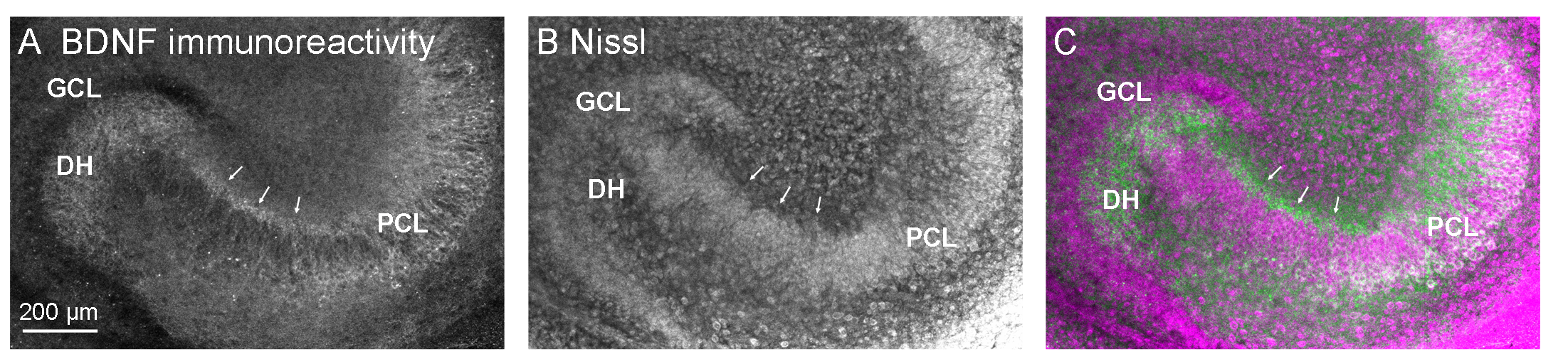

Supplement: Additional file 1 — Endogenous BDNF was expressed primarily along the mossy fiber pathways in cultured hippocampal slices, as observed in brain sections. Merged images of hippocampal slices stained (C, merged) with both Nissl staining of neurons (B) and BDNF immunohistochemistry (A). BDNF was highly expressed in the SL (arrow) and the dentate hilus, which are the normal mossy fiber pathways. GCL: granule cell layer, PCL: pyramidal cell layer, and DH: dentate hilus. [file 1756-6606-2-2-S1.tiff]
